# Unified Protocol for the Transdiagnostic Treatment of Emotional Disorders in Group Format in Spain: Results of a Noninferiority Randomized Controlled Trial at 15 Months after Treatment Onset

**DOI:** 10.1155/2023/1981377

**Published:** 2023-06-30

**Authors:** O. Peris-Baquero, J. Osma

**Affiliations:** ^1^Psychology and Sociology Department, University of Zaragoza, Teruel 44003, Spain; ^2^Health Research Institute of Aragon, Zaragoza 50009, Spain

## Abstract

Evidence-based psychological treatments (EBPT) are an effective and efficient solution for the treatment of emotional disorders (EDs). However, their implementation and dissemination are not yet widespread. The Unified Protocol for Transdiagnostic Treatment of EDs (UP), applied in a group format, could be an effective option to be implemented in specialized public mental health services in Spain. The sample consisted of 533 users of public specialized mental health centers (77.3% women), with a mean age of 42.0 years (SD = 12.62), who were randomized to the UP in group format condition (*n* = 277) or treatment as usual (specific cognitive behavioral therapy for each disorder in individual format, *n* = 256). Assessments were performed at preintervention (T1) and at 3, 6, 9 and 15 months after treatment onset (T2, T3, T4, and T5, respectively). The results showed a main effect of time in both conditions for all primary outcomes (*p* < 0.05) and no statistically significant Time⁣^∗^Condition interaction. Similarly, the noninferiority tests showed that UP results were statistically noninferior compared to TAU. Effect sizes for psychological variables were higher in the UP condition at T5, even though the differences were not statistically significant. Statistically significant differences (*p* < 0.05) in the evolution of the diagnostic criteria and comorbidity were found, with the highest percentage of patients no longer meeting main and secondary diagnosis criteria in the UP condition at all assessment moments (except for secondary diagnosis at T3). The results showed statistically significant differences in treatment retention between conditions at T5, being the UP condition the one with less dropouts. Finally, participants in the group UP condition showed high satisfaction with the treatment. The UP is an EBPT that has been shown to be effective when applied in groups and may represent an efficient option for its implementation in public mental health services in Spain. This trial is registered with NCT03064477.

## 1. Introduction

Evidence-based practice is defined as “the professional practice of psychology based on empirically supported criteria and integrated with the therapeutic relationship in the context of the patient's characteristics, culture and preferences” [1]. In this regard, a treatment is considered empirically supported if it has demonstrated its efficacy, through randomized clinical trials (RCTs) and systematic reviews or meta-analyses [2]. Consequently, the use of evidence-based psychological treatments (EBPT) is aimed at improving the quality of the psychological services offered [3].

Some of the advantages offered by the EBPT are that these treatments have proven to be effective and are also protocolized, which facilitates their dissemination and application [4]. In addition, these treatments can be format adapted to optimize the resources of the context in which they are applied; this is the case of the group format that allows clinicians in public mental health settings to attend a greater number of patients in a shorter period of time. However, the literature shows discrepancies on the effectiveness of the format, with different pieces of evidence in favor of individual format [5, 6], equal effectiveness between the two formats [7, 8], or in favor of the individual or group format depending on the severity symptoms of the participants and the preferences of choice [9].

Along with the advantages and closely related to the formats of intervention, some EBPT are also transdiagnostic, which allow intervention focused on mechanisms that are shared by several disorders [10], something very useful for the treatment of comorbidity, which is highly prevalent in psychological disorders [11]. Specifically, it is estimated that the lifetime rate of comorbidity between depressive and anxiety disorders is around 75% [12]. Moreover, this is especially important given that diagnostic comorbidity is related to greater symptomatologic severity [13, 14]. For this reason, the transdiagnostic approach is effective in reducing not only the specific symptoms of each disorder but also the symptoms of comorbid diagnoses [15]. Moreover, this transdiagnostic approach would reduce the high costs derived from the training of psychologists in the different treatments for each disorder, as well as reduce the costs derived from their implementation [16]. This is especially important for public health systems, since, if we look at data from Spain, the cost associated with dealing with mental health problems is around 4.2% of gross domestic product (GDP) [17].

However, despite the advantages offered by EBPTs, their inclusion in clinical practice is still very limited [18]. For example, it has been proved that only one in six people diagnosed with a major depressive disorder received effective treatment [19] and around 28-36% for anxiety disorders [20, 21]. Also alarmingly, in Spain, it was found that 43% of people with depression, or 70% of people with anxiety, did not receive psychological treatment [22]. For this reason, promoting access to EBPT remains a key objective, as set out in the WHO European Framework for Action on Mental Health 2021-2025 [23].

The use of EBPT is critical in public health systems where resources are limited [24] and specially for addressing the most prevalent disorders. If we look at prevalence data, we find that anxiety and mood disorders (which can be included in the so-called emotional disorders (EDs) ([25])) are the most prevalent [26]. According to the European Health Survey 2020, around 5.8% of the population in Spain suffers from anxiety and around 5.4% from depression [27]. Finally, it should be noted that EDs have become the leading cause of burden worldwide [28].

A transdiagnostic EBPT specifically designed for the treatment of EDs is the Unified Protocol for the Transdiagnostic Treatment of Emotional Disorders (UP; [29, 30]). The transdiagnostic nature and versatility of this treatment allow it to be applied in different formats, including in group [31, 32] or online [33].

Regarding the effectiveness of the UP, to date, several studies have analyzed the efficacy of the UP [34–36] founding moderate to large effect sizes in the reduction of ED symptomatology (Hedges' *g* between 0.45 and 1.11). In addition, the UP has also shown good results in terms of acceptability by the psychologists involved in the treatment, with overall acceptability values of 4.30 out of 5 (SD = 0.68, range 3-5) and intention to use in the future 4.54 out of 5 (SD = 0.56, range=3-5) [37].

Despite these promising results, there are still very few studies that have included long follow-up periods with the aim of analyzing whether improvements in the symptomatology of EDs were maintained over time. To the best of our knowledge, only two studies have applied the UP in a group format in a naturalistic context reporting long-term outcomes. The first one, conducted in Denmark by Reinholt et al. [32], which included 291 patients who received the UP applied in a group format, showed that after 6 months of follow-up, the UP group was not inferior to the dCBT condition in the improvement of depression (mean difference between conditions 0.20; 95% CI -1.01 to 1.42; *p* = 0.74, Cohen's *d* = 0.05, -0.24 to 0.34) and anxiety symptoms (mean difference between conditions 0.69; 95% CI -0.61 to 1.99; *p* = 0.30, Cohen's *d* = 0.15, -0.14 to 0.44). The second study was carried out by our research team and analyzed the efficacy of the UP applied in a group format after 6 months of follow-up, obtaining greater changes in depression, anxiety, and quality of life in the group UP condition (Cohen's *d* between 0.69 and 0.90) compared to TAU in individual format (Cohen's *d* between 0.41 and 0.70) [31].

Therefore, although the UP is accumulating evidence of its efficacy, there are still limited results regarding its efficacy in group format and especially in naturalistic contexts (i.e., in public health systems) that include long-term data. Considering the aforementioned evidence, the aim of the present study is to analyze the long-term efficacy of the UP applied in group format in Spanish public mental health units and to compare its results after 15 months after treatment onset (T5) with the treatment habitually used in the public health system. In addition, we will compare the treatment retention, the evolution of diagnostic comorbidity in both conditions, and the satisfaction with the treatment of the participants in the UP condition in group format. Considering the previous studies, and given that this is a noninferiority study, we hypothesize that the UP applied in group format will achieve statistically significant changes in the variables under study and that the results will be statistically noninferior to those obtained by the usual treatment condition after 15 months after treatment onset (T5) in primary and secondary outcomes. We also expect to achieve similar treatment retention to that of the usual condition, a reduction in diagnostic comorbidity, and finally high satisfaction with the treatment by the participants in the group UP condition.

## 2. Materials and Methods

### 2.1. Study Design

The present study is a multicenter, noninferiority randomized (with balanced randomization (1 : 1) and stratified according to the participants' severity based on their scores in anxious and depressive symptoms, assessed by the BDI-II and BAI), single-blinded, parallel, controlled trial [38]. This trial is registered with NCT03064477 (ClinicalTrials.gov). The study report followed CONSORT (Consolidated Standards of Reporting Trials) guidelines [39]. All participants completed and signed the informed consent form, and the study was approved by the Research Ethics Committees of the collaborating centers (for more information, see [38]).

### 2.2. Participants

**Figure 1:**
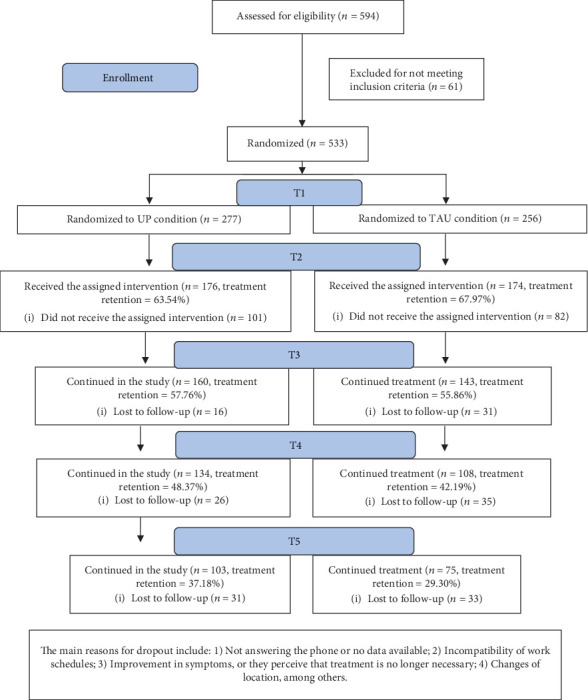
Flowchart of study participants following the CONSORT guidelines.

A total of 533 people (77.3% female) with a mean age of 42.0 years (SD = 12.62, range=18-77) participated in this study, and all of them were included in the intention-to-treat analysis.  Figure 1 shows the flowchart of the participants according to the CONSORT guidelines. Participants with a primary diagnosis of EDs and who met the inclusion criteria were randomized to two active treatment conditions: Unified Protocol applied in group format (UP condition: *n* = 277, mean age = 42.04, SD = 11.93, range=18-70) and treatment as usual in individual format (TAU condition: *n* = 256, mean age = 41.93, SD = 13.22, range=18-77). The rest of the sociodemographic information can be found in Table 1.

### 2.3. Instruments

#### 2.3.1. Primary Outcomes

*Sociodemographic Information.* Ad hoc questionnaire was used to collect information on participants' age, sex, level of education, marital and job status, primary and secondary diagnoses, and psychotropic medication use.

*Clinical Diagnoses*. Anxiety Disorders Interview Schedule (ADIS-IV; [40]), semistructured interview for the evaluation and diagnosis of anxiety and depressive disorders, and others such as somatoform and substance use disorders according to DSM-IV criteria [41]. We could not use the ADIS-5 [42] because is not yet available in Spanish.

*Depression*. Depression was assessed through the Beck Depression Inventory (BDI-II; [43, 44]). Through 21 items, it evaluates the severity of depressive symptomatology with a response scale of 4 options ranging from 0 (least severity of symptomatology) to 3 (greatest severity). The Overall Depression Severity and Impairment Scale was also used (ODSIS; [45, 46]), which evaluates the interference, frequency, and intensity of depressive symptomatology in the previous week through 5 items with a response scale from 0 “not at all” to 4 “completely.” Cronbach's alpha of both instruments in the present sample was 0.91 and 0.94, respectively.

*Anxiety*. Anxiety was assessed through the Beck Anxiety Inventory (BAI; [47, 48]). Through 21 items, it evaluates the severity of the anxious symptomatology with a response scale of 4 options ranging from 0 (least severity of symptomatology) to 3 (greatest severity). The Overall Anxiety Severity and Impairment Scale was also used (OASIS; [46, 49]), which evaluates the interference, frequency, and intensity of anxious symptomatology in the previous week through 5 items with a response scale from 0 “not at all” to 4 “completely.” Cronbach's alpha of both instruments in the present sample was 0.92 and 0.87, respectively.

#### 2.3.2. Secondary Outcomes

*Personality*. The dimensions of neuroticism and extraversion were assessed through the 24 items of the NEO Five-Factor Inventory (NEO-FFI; [50]). This measure uses a Likert-type response scale ranging from 0 “strongly disagree” to 4 “strongly agree.” The internal consistency in our sample was *α* = 0.76 for neuroticism and *α* = 0.80 for extraversion.

*Affect.* Positive and negative affect was assessed through the Positive and Negative Affect Schedule (PANAS; [51, 52]). This scale is composed of 20 items and uses a 5-point Likert-type response scale ranging from 1 “not at all or almost not at all” to 5 “very much.” Cronbach's alpha was *α* = 0.90 for the positive affect dimension and *α* = 0.92 for the negative affect in our sample.

*Quality of Life*. Self-perceived quality of life was assessed through the Quality of Life Index (QLI; [53]), which consists of 10 items and evaluates different vital dimensions through a 10-point Likert-type response scale ranging from 0 “poor” to 10 “excellent.” The internal consistency of QLI was *α* = 0.87.

#### 2.3.3. UP Intervention Satisfaction and Utility Assessment

*Treatment Satisfaction Questionnaire* (STQ; adapted from the Client Satisfaction Questionnaire [CSQ-8] de [54]). We have adapted the CSQ-8 to include 6 of the 8 items (perceived quality, appropriateness to previous expectations, recommendation of the treatment to friends or family, usefulness of the techniques learned, overall satisfaction with the intervention, and likelihood of choosing this type of intervention again) and one more item related to the discomfort generated by the intervention. Likewise, the Likert response scale has been changed from 4 points in the original from 0 “bad/nothing” to 4 “excellent/very much” to 11 in the current one from 0 “bad/nothing” to 10 “excellent/very much.”

*Unified Protocol Module Evaluation Questionnaire*. Ad hoc questionnaire is comprised of 6 questions that separately evaluate the usefulness, difficulty, and satisfaction of each of the techniques used in the different modules of the UP. The response scale is Likert-type and ranges from 0 “not at all” to 10 “very much.”

### 2.4. Procedure

Participants in this study were recruited from different public mental health units of Spain collaborating in the clinical trial [38]. Recruitment was conducted from March 2018 to December 2019, with 42 collaborating professionals from 19 public mental health units. All those users of the public health system who attended their different referral centers and who presented a main diagnosis of EDs, evaluated through the ADIS-IV clinical interview [40], were proposed as potential candidates by the clinical psychologists collaborating in the project. The inclusion criteria were the following: (a) main diagnosis of EDs (e.g., anxiety disorder, mood disorder, and related disorders); (b) to be over 18 years of age; (c) to fully understand the language in which the therapy is conducted; (d) to be able to participate in the evaluation and treatment sessions and sign the informed consent; and (e) in case of pharmacological treatment, to maintain it unchanged during the 3 months preceding the beginning and during the psychological treatment. The exclusion criteria were as follows: (a) presence of a severe mental disorder (e.g., bipolar disorder, schizophrenia, or organic mental disorder) and current risk of suicide or substance abuse in the previous 3 months (excluding cannabis, coffee, or nicotine use) and (b) the patient has received 8 or more sessions of psychological treatment based on the principles of cognitive behavioral therapy in the last 5 years.

Those participants who finally met the inclusion criteria were invited to participate in the project and to sign the informed consent form. Once the selection process and the initial assessment battery had been completed, an external researcher carried out a stratified randomization of the participants. For this purpose, participants were grouped according to their severity based on their scores in anxious and depressive symptomatology (assessed by the BDI-II and BAI). Next, they were randomized through a computer software (Randomizer), to each of the two treatment conditions. This was done so that the distribution of the participants' symptomatologic severity would be equal between the two conditions.

Concerning the treatment conditions, on the one hand, there was the UP condition in group format, which consisted of 12 treatment sessions applied in group format and based on the 8 original treatment modules of the UP manual ([29]; for more information, see [38]). The sessions were applied over 2 hours in a weekly basis for approximately 3 months by a therapist and a cotherapist, previously trained in UP. On the other hand, there was TAU condition; this consisted of nonprotocolized cognitive behavioral treatment for each specific disorder applied in individual format, with the usual number of sessions, frequency of appointments, and duration of the sessions of each mental health unit. The details of both interventions can be consulted in detail in the study protocol [38]. Finally, evaluations were carried out over a period of 15 months as follows: T1 (preintervention assessment), T2 (3 months after treatment onset and coinciding with the end of the UP intervention), T3 (6 months after treatment onset), T4 (9 months after treatment onset), and T5 (15 months after treatment onset). Due to the difficulties of conducting controlled studies in public health care settings, it was only possible to readminister the diagnostic interview to a percentage of the sample after treatment onset.

### 2.5. Data Analysis

All participants randomized to the treatment conditions were included in the intention-to-treat analyses. First, descriptive statistical analyses were carried out to analyze the characteristics of the study participants. Once this was done, comparisons between groups were carried out through ANOVA for continuous variables and chi-square analysis for categorical variables.

Next, a mixed model analysis was carried out, specifically a linear mixed model analysis which included as main effects the variable “Time,” “Treatment condition,” and “number of sessions.” The variable “Center” was also included as random effects to analyze whether there were differences in the evolution of the scores depending on the center where the intervention had been carried out. The main effects of time, as well as the interaction “Time⁣^∗^Condition” and “Time⁣^∗^Condition⁣^∗^Sessions” were analyzed with the aim of analyzing whether there was a different evolution of scores over time due to the treatment condition and/or the number of sessions received. For those results where statistically significant main or interaction effects were found, post hoc analyses were performed. Specifically, Pearson's *R* correlation or linear mixed models were replicated by segmenting the participants according to treatment condition and grouping them according to the number of sessions received (8 to 12 or more than 12 in the UP condition and less than 8 or 8-12 in the TAU condition). Data from less than 8 sessions in the UP condition and more than 12 sessions in the TAU condition were not included in the post hoc analyses due to the small number of participants with these characteristics.

Finally, noninferiority analyses were carried out according to the recommendations for this type of study [55]. A minimum sample size required for noninferiority analyses of 95 participants was established in each group with a power of 80%, an alpha level of 0.05 (one-sided), a standard deviation of 0.83 in the CBT group compared to placebo [56]. The noninferiority margin was defined a priori by a strict and small margin of tolerance for noninferiority of 10%, in accordance with recommendations [57, 58], applied to the lower bound when the UP showed smaller improvements in comparison to active control condition or to the upper bound of equivalence of the TOST equivalence test based on the results obtained in the UP efficacy meta-analysis over active control condition (*g* = 380; 95% CI of 0.094 to 0.666; [34]). In addition, to ensure noninferiority, a per-protocol analysis was conducted following FDA guidelines to reduce the possibility of bias [59]. Finally, the analyses included calculation of effect size (Cohen's *d*), interpreted as small (*d* ≈ 0.2), medium (*d* ≈ 0.5), or large (*d* ≈ 0.8). All statistical analyses were carried out using the statistical software SPSS version 25.0 [60], except for the noninferiority analyses, which were performed with Jamovi 2.2.5 [61] and the TOSTER module [62].

## 3. Results

### 3.1. Sociodemographic Results, Reduction of Symptoms Severity, Evolution of Diagnostic Criteria and Comorbidity, and Treatment Retention between Groups

Analyses of comparison of means (ANOVA) showed no statistically significant differences at baseline in any of the study variables (*p* > 0.05), nor were differences found in the sociodemographic variables of gender (*χ*^2^(1) = 0.00, *p* = 0.944), primary diagnosis (*χ*^2^(2) = 0.13, *p* = 0.935), secondary diagnosis (*χ*^2^(20) = 12.30, *p* = 0.905), and psychotropic medication usage (*χ*^2^(1) = 0.46, *p* = 0.498). Regarding the number of sessions received, in the UP condition, the mean number of sessions received at T2 was 8.25 (SD = 3.91, range 1-14), at T3 was 11.46 sessions (SD = 3.91, range 7-16), at T4 was 12.50 sessions (SD = 2.67, range 7-19), and at T5 was 13.65 sessions (SD = 2.61, range 7-20). In addition, 13.6% (*n* = 38) of the participants in the UP condition required extra treatment sessions in individual format, specifically a mean of 2.74 sessions (SD = 2.06, range 1-6). For participants in the TAU condition, the mean number of sessions received at T2 was 2.24 (SD = 1.66, range 1-6), at T3 was 3.97 sessions (SD = 3.97, range 2-10), at T4 was 5.54 (SD = 2.40, range 2-12), and at T5 was 7.69 sessions (SD = 3.03, range 2-15).

Concerning the reduction of symptom severity, specifically for the BDI-II, mean scores improved from T1 to T2 by an average of 78.4% in the UP condition and 77.2% in the TAU condition and from T1 to T5 by an average of 86.4% in the UP condition and 74.3% in the TAU condition. For BAI, mean scores improved from T1 to T2 by an average of 73.1% in the UP condition and 61.4% in the TAU condition and from T1 to T5 by an average of 78.8% in the UP condition and 75.7% in the TAU condition. Using the cutoff severity score of 20 on BDI, 55.7% participants in the UP condition presented scores below moderate at T2, versus 45.6% of the TAU participants, and 66.0% of the UP condition participants versus 58.6% of the TAU condition at T5. For anxious symptoms and using a severity cutoff score of 16 on the BAI, 39.4% of UP condition participants had scores below moderate at T2, versus 36.0% of TAU participants, and 57.7% of UP condition participants versus 51.1% of TAU condition participants at T5.

Regarding the evolution of the diagnostic criteria and comorbidity, as can be seen in Table 2, statistically significant differences were found between conditions (*p* < 0.05), with the highest percentage of patients no longer meeting the criteria of the main diagnosis in the participants of the UP condition at all assessment moments. A similar result was observed in the evolution of the secondary diagnoses, with statistically significant differences between groups and with the highest percentage of participants no longer meeting criteria for the secondary diagnosis in the UP condition (except at time T3, where no statistically significant differences (*p* > 0.05) were found between conditions).

Finally, the treatment retention in each treatment condition can be found in Figure 1. The results showed statistically significant differences in treatment retention between conditions at T5 (*χ*^2^(1) = 5.88, *p* = 0.015), being the UP condition the one with less dropouts.

### 3.2. Main Effects and Random Effects of the Linear Mixed Models

As we can see in Table 3, the linear mixed models showed a main effect of time with statistically significant changes in the BDI-II, ODSIS, BAI, OASIS, NEO-FFI_neuroticism, PANAS_negative affect, and QLI. However, no main effects of time were found on the NEO-FFI_extraversion and PANAS_positive affect. No main effects of treatment condition were found either (*p* > 0.05). Finally, the results showed a main effect of the number of sessions on PANAS_positive affect, showing a positive relationship between the greater number of sessions received with higher scores on PANAS_positive affect (*r* = 0.230, *p* = 0.010).

Regarding the random effects of the variable “center,” the results showed a statistically significant effect on the variable NEO-FFI_neuroticism (Wald *Z* = 2.24, *p* = 0.025, 95% confidence interval: 0.63-3.62) and PANAS_negative affect (Wald *Z* = 3.01, *p* = 0.003, 95% confidence interval: 2.68-9.85). Specifically, the results showed statistically significant effects of time on NEO-FFI_neuroticism, except in 5 of the centers where UP was applied and in 5 of the centers where TAU was applied. With respect to PANAS_negative affect, no statistically significant effect of time was found in 6 centers for the TAU condition and in 6 centers for the UP condition. For the variables ODSIS, OASIS, and QLI, no statistically significant random effects of the variable “Center” were found (*p* > 0.05), and for the NEO-FFI_extraversion, PANAS_positive affect, BDI-II, and BAI, convergence issues were found, despite increasing the number of interactions (up to 3000).

### 3.3. Interaction Effects of the Linear Mixed Models and Noninferiority Results

In terms of the interaction effects, and as can be seen in Table 3, no statistically significant Time⁣^∗^Condition interaction effects were found in any of the study outcomes (*p* > 0.05), so that both conditions were comparable in the evolution of the scores. However, if we analyze in depth the results in each condition, and as can be seen in Table 4, the results showed statistically significant changes in the UP condition in all study outcomes. Similar results were obtained in the TAU condition, except for the NEO-FFI_extraversion, where no statistically significant changes were found.

Considering the effect sizes in both treatment conditions, we found that when comparing changes between T1 and T5, the UP condition obtained higher effect sizes in all variables with medium-high scores (Cohen's *d* between 0.36 and 1.03), while the TAU condition obtained small-moderate effect sizes (Cohen's *d* between 0.16 and 0.72).

In addition, as we can see in Table 5, the results showed statistically significant differences between treatment conditions at T2 in the variable ODSIS and at T3 in ODSIS, BAI, OASIS, PANAS_negative affect, and QLI and statistically significant differences at T3 in the variables OASIS and QLI. All differences were in favor of the UP condition. Finally, no statistically significant differences were found between treatment conditions at T5 (*p* > 0.05). Similarly, the noninferiority tests showed that UP results were statistically noninferior compared to TAU in all variables except for BAI, neuroticism, negative affect, and quality of life comparing T1 with T5 (*p* > 0.05), where the greatest improvements were found in the UP condition.

Finally, the linear mixed models showed a Time⁣^∗^Condition⁣^∗^Sessions interaction effect for ODSIS (Table 3). Post hoc analyses conducted by classifying participants according to treatment condition and number of sessions received showed a main effect of time in UP condition participants who received between 8 and 12 sessions and more than 12 sessions, as can be seen in Table 6. On the other hand, a main effect of time was also found for participants in the TAU condition who received less than 8 treatment sessions, but not so for participants who received between 8 and 12 sessions. As for effect sizes, participants in the UP condition showed moderate-large effect sizes (Cohen's *d* between 0.77 and 1.57), with the highest value corresponding to participants who received between 8 and 12 sessions, whereas participants in the TAU condition showed small-moderate effect sizes (Cohen's *d* between 0.06 and 0.73).

### 3.4. Evaluation of the Satisfaction and Utility of the UP Intervention

The results from the satisfaction with the intervention showed that participants rated the intervention positively, with a general mean score of 8.41 (SD = 1.17, range 4-10); the “Mindful emotion awareness” module was the highest rated (*M* = 9.00, SD = 1.56, range 4-10), while the “Understanding and confronting physical sensations” was the lowest rated (*M* = 7.20, SD = 3.32, range 0-10). Participants indicated that “Understanding the adaptability of emotions” and “Mindful emotion awareness” modules were the most useful (*M* = 9.47, SD = 0.9, range 7-10), while the “Understanding and confronting physical sensations” module scored the lowest (*M* = 8.47, SD = 1.84, range 5-10). In terms of difficulty, the “Exposure to emotions” module was rated as the hardest (*M* = 8.07, SD = 2.60, range 0-10), while the “Understanding the adaptability of emotions” module was the least difficult (*M* = 5.27, SD = 3.35, range 0-10).

## 4. Discussion

This is the first multicenter, randomized, noninferiority clinical trial that has analyzed the long-term efficacy of UP applied in group format in public mental health units in Spain. Our first hypothesis was that the UP treatment applied in a group format would achieve statistically significant changes in the variables under study and that the results would not be inferior to those obtained by the TAU after 15 months after treatment onset (12 months after the end of the UP intervention). The results obtained in this study have confirmed our hypothesis.

Regarding the efficacy results, as we have seen, both interventions produced statistically significant changes in the primary outcomes, specifically reductions in depressive and anxious symptomatology and improvements in quality of life, with small effect sizes (Cohen's *d* = 0.21 to 0.28). In addition, interaction effects showed that there was no Time⁣^∗^Condition interaction effect and the noninferiority tests showed that UP results were statistically noninferior compared to TAU, so both interventions were comparable, and these results were consistent with other studies in a similar context comparing two active treatment conditions (i.e., [32, 63]).

In addition to changes in the primary outcomes, the results also showed changes in other outcomes closely related to the mechanisms associated with the etiology and maintenance of EDs, such as neuroticism or negative affect, with small to medium effect sizes (Cohen's *d* = 0.17 to 0.31). These results were similar to those obtained by other studies [64–66]. However, no statistically significant changes were obtained in the variables of extraversion and positive affect, considering the combined efficacy of both interventions.

Although the “Time⁣^∗^Condition” interaction results did not show statistically significant effects, if we consider the results of both interventions separately, it is necessary to highlight several outcomes. First, in relation to the reduction of symptomatologic severity, the results obtained show a higher percentage of recovery in the UP group compared to the TAU; this is also reflected in the reduction of diagnostic comorbidity, being statistically superior in the UP condition. These results coincide with the existing literature and support the transdiagnostic efficacy of the intervention [15, 34]. Secondly, the effect sizes achieved at T5 were higher for all variables in participants in the UP condition, with medium to large effect sizes (Cohen's *d* 0.36 to 1.03) versus the TAU condition, with small to moderate effect sizes (Cohen's *d* 0.16 to 0.72). In addition, there were statistically significant changes in all variables in the UP condition, including extraversion, with medium effect sizes (Cohen's *d-*0.36 at T5), in contrast to the TAU condition, where no statistically significant effect was found in this variable. This finding is noteworthy since there are contradictory results about changes in extraversion and positive affect after the application of the UP, and in our case, it coincides with the results obtained by other studies such as the one carried out by Grill et al. [67] or Reinholt et al. [68]. The communalities shared by these three studies is that neither of them included a specific module to enhance positive affect and all of them used the group format delivery. In this sense, the group intervention format may have favored these results [69] allowing participants to put their social skills into practice in a much more explicit way than in other types of formats such as individual or online, encouraging empathy, support, commitment, and positive ways of relating and working together in therapeutic groups [70]. Considering these results, it is possible that no complementary contents regarding positive affect are necessary to achieve changes in these variables.

Moreover, it is important to note that the greatest changes in the UP condition occurred in the first 3 months of the intervention and that the results were maintained up to T5 (15 months after treatment onset). This is especially important because, as shown by the mean comparison results, there were no statistically significant differences at T5 between the UP condition and the TAU condition. Then, although participants in the TAU condition had continued to receive active treatment sessions over time, participants in the UP condition had completed the intervention one year earlier. Considering our results, it seems that through a brief and intensive UP group intervention (applied in only 3 months), we can obtain statistically significant changes in the emotional symptomatology and ED's vulnerability factors of the patients and maintain it in the long term. Some research and clinical implications can be discussed based on these results. A greater number of feasibility and implementation studies are still needed to analyze the best possible adaptions of brief intensive transdiagnostic group interventions to specific contexts (i.e., specialize mental health settings, primary care units, community settings, etc.) and to study implementation barriers and the acceptability and intention to use the interventions in the future by clinicians, among other important topics [37, 71]. Furthermore, policymakers, mental health providers, and directors of clinical psychology training programs for residents should provide specific training in transdiagnostic interventions and group format applications for clinicians. All these needs require to increase human and economic resources in mental health, and doing this, it will be possible to improve public health, economic welfare, and social equity [72].

Another aspect that should be mentioned is the difference in the number of sessions received. The results of this study have shown that, although a greater number of sessions seems to be associated with higher positive affect scores, only a statistically significant interaction effect was found in ODSIS. The results of the post hoc analysis showed that the greatest changes occurred when participants in the UP condition received between 8 and 12 treatment sessions. In addition, a greater number of sessions did not show major changes. This result would suggest that a reduced number of sessions would be sufficient to produce improvements in the participants. Furthermore, according to the results of this study, only 13.6% (*n* = 38) of the participants required extra treatment sessions, so the way the treatment is structured would be sufficient for most of the patients. Finally, thanks to its application in group format, it could be a more cost-effective format for the health system than the individual format, as has been shown in a recent publication [73]. In addition, it should be noted that the NICE (National Institute for Health and Care Excellence) guidelines recommend an average of 8 weekly sessions for psychological treatment to be considered effective [74], so this intervention format would allow to comply with this recommendation.

Regarding treatment retention, the results have shown a similar treatment retention at T2 in both conditions, although the group format is not usually the format of choice for mental health users [75]. However, it is necessary to mention that while 63% of the participants assigned to the UP condition have completed the treatment (with an average of 8 sessions), the participants in the TAU condition have had only an average of 2 treatment sessions and have not yet completed the treatment. It is necessary to wait until the T5 (15 months after treatment onset) for participants in the TAU condition to receive an average of 8 treatment sessions. However, only 29.30% of the participants in this condition continue in the treatment until this time. On this regard, it is important to highlight the length of time that patients are suffering in our mental health system until they receive a minimum recommended number of sessions (approximately 8 sessions, [74]).

Finally, the results have shown high overall patient satisfaction with the UP treatment (8.41 out of 10, SD = 1.17, range 4-10), as well as high usefulness and low difficulty of the different modules that make up the treatment. It should be noted that satisfaction with the intervention received is directly associated with efficacy and adherence to treatment [76].

Despite the promising results, this study also has several limitations that should be mentioned. First, this study has been carried out in a public context in mental health units in Spain, so the results of this study may not be applicable to other contexts and to other patient profiles than those treated in the public system. Furthermore, the situation of the public health systems and the difficulty of having enough time to carry out complete diagnostic interviews made it difficult to readminister the diagnostic interview after the start of treatment, which meant an important loss of information for many of the participants in this study. In addition, the TAU condition does not follow a structured and protocolized treatment, and the number of sessions is highly variable, making it difficult to compare interventions. However, one of the strengths of this study is that it has been implemented in a naturalistic context. In addition, to our knowledge, this is the RCT with the largest sample that has analyzed the efficacy of UP compared to an active condition. Another limitation is that we found differences in the results depending on the center where the interventions were applied. In future studies, it will be necessary to include a greater number of supervision mechanisms to analyze adherence to the UP treatment and to analyze in depth the techniques and tools used by the therapists in the TAU condition. Finally, most of the participants in this study were women (77.3%), which may also affect the generalizability of the results. Although the prevalence of EDs is more than twice as high in women as in men, it is not possible to generalize the results [77]. Future studies should analyze these differences. Finally, future studies should analyze whether there are differences in efficacy in the UP depending on the application format (group or individual) and analyze whether there are profiles that can benefit more from one intervention format or another, adapting the intervention to their characteristics, as suggested by other studies [9].

## 5. Conclusions

The results obtained in this study have shown that a brief UP intervention applied in a group format over 3 months can be a feasible, acceptable, and efficient intervention for the Spanish public mental health system. UP has achieved improvements in emotional symptomatology, in ED vulnerability factors, including extraversion and positive affect, and in quality of life, being these results statistically noninferior to those achieved by the TAU treatment (specific treatment of each specific disorder), and those improvements have been maintained until 15 months after treatment onset. Clinicians offering UP groups to treat their patients with EDs in public mental health settings can provide an EBPT to a greater number of patients, who will benefit from the reduction of clinical symptoms in a short period of time (approximately 3 months), reducing the time of suffering of people with EDs and maintaining the benefits of the intervention in the long term. The UP has previously demonstrated high scores on acceptability and intention to use the UP in future by clinicians working in public mental health settings in Spain [37], and through the present study, high satisfaction scores have been obtained from patients. To sum up, the results of this study can contribute to the dissemination of the UP, a transdiagnostic EBPT for EDs, in naturalistic mental health settings.

## Figures and Tables

**Table 1:** Sociodemographic characteristics and primary and secondary diagnoses of the participants (*N* = 533).

	UP (*n* = 277)	TAU (*n* = 256)	Total (*N* = 533)
	*n* (%)	*n* (%)	*n* (%)
Educational level			
More than 12 years of education	170 (61.4)	154 (60.2)	324 (60.8)
University studies	73 (26.4)	61 (23.8)	134 (25.1)
Vocational training	70 (25.3)	64 (25.0)	134 (25.1)
High school	27 (9.7)	29 (11.3)	56 (10.5)
Less than 12 years of education	107 (38.6)	102 (39.8)	209 (39.2)
Primary studies or less	56 (20.2)	59 (23.0)	115 (21.6)
Secondary studies	51 (18.2)	43 (16.8)	94 (17.6)
Marital status			
Married/living with partner	143 (51.6)	132 (51.6)	275 (51.6)
Not married/not living with partner	134 (48.4)	124 (48.4)	258 (48.4)
Single	80 (28.9)	90 (35.2)	170 (31.9)
Separated/divorced	46 (16.6)	29 (11.3)	75 (14.1)
Widowed	8 (2.9)	5 (2.0)	13 (2.4)
Job status			
Not working	157 (56.7)	145 (56.6)	302 (56.7)
Unemployed	55 (19.9)	61 (23.8)	116 (21.8)
Sick leave	55 (19.9)	44 (17.2)	99 (18.6)
Student	24 (8.7)	19 (7.4)	43 (8.1)
Homemaker	15 (5.4)	13 (5.1)	28 (5.3)
Retired	8 (2.9)	8 (3.1)	16 (3.0)
Working	120 (43.3)	111 (43.4)	231 (43.3)
Primary diagnoses			
Anxiety and related disorders	131 (47.3)	117 (45.7)	248 (46.5)
Generalized anxiety disorder	31 (11.2)	29 (11.3)	60 (11.3)
Panic disorder with agoraphobia	26 (9.4)	20 (7.8)	46 (8.6)
Non-specific anxiety disorder	17 (6.1)	20 (7.8)	37 (6.9)
Panic disorder without agoraphobia	15 (5.4)	14 (5.5)	29 (5.4)
Agoraphobia	16 (5.8)	7 (2.7)	23 (4.3)
Obsessive-compulsive disorder	10 (3.6)	11 (4.3)	21 (3.9)
Hypochondria	7 (2.5)	5 (2.0)	12 (2.3)
Social anxiety	4 (1.4)	6 (2.3)	10 (1.9)
Traumatic stress disorder	5 (1.8)	5 (2.0)	10 (1.9)
Mood disorders	81 (29.2)	71 (27.7)	152 (28.5)
Major depressive disorder	53 (19.1)	50 (19.5)	103 (19.3)
Dysthymia	20 (7.2)	14 (5.5)	34 (6.4)
Unspecified mood disorder	8 (2.9)	7 (2.7)	15 (2.8)
Mixed disorders	65 (23.5)	68 (26.6)	133 (25.0)
Adjustment disorder	65 (23.5)	68 (26.6)	133 (25.0)
Secondary diagnoses			
Anxiety and related disorders	52 (18.8)	36 (14.1)	88 (16.5)
Non-specific anxiety disorder	13 (4.7)	13 (5.1)	26 (4.9)
Generalized anxiety disorder	13 (4.7)	5 (2.0)	18 (3.4)
Agoraphobia	8 (2.9)	4 (1.6)	12 (2.3)
Obsessive-compulsive disorder	4 (1.4)	5 (2.0)	9 (1.7)
Panic disorder without agoraphobia	3 (1.1)	4 (1.6)	7 (1.3)
Social anxiety	6 (2.2)	1 (0.4)	7 (1.3)
Hypochondria	2 (0.7)	2 (0.8)	4 (0.8)
Panic disorder with agoraphobia	2 (0.7)	1 (0.4)	3 (0.6)
Traumatic stress disorder	1 (0.4)	1 (0.4)	2 (0.4)
Mood disorders	20 (7.2)	20 (7.8)	40 (7.5)
Major depressive disorder	14 (5.1)	13 (5.1)	27 (5.1)
Dysthymia	6 (2.2)	6 (2.3)	12 (2.3)
Unspecified mood disorder	—	1 (0.4)	1 (0.2)
Mixed disorders	6 (2.2)	6 (2.3)	12 (2.3)
Adjustment disorder	6 (2.2)	6 (2.3)	12 (2.3)
Psychotropic medication			
Taking psychotropic medication	200 (72.2)	178 (69.5)	378 (70.9)

Note: UP: Unified Protocol; TAU: treatment as usual.

**Table 2:** Frequency and differences between groups in diagnostic comorbidity over time.

			T2			T3			T4			T5		
			*n* (%)	*χ* ^2^	*p*	*n* (%)	*χ* ^2^	*p*	*n* (%)	*χ* ^2^	*p*	*n* (%)	*χ* ^2^	*p*
Main diagnosis	Meets diagnostic criteria	UP	53 (62.4)	**5.98**	**0.014**	25 (41.7)	**19.56**	**≤0.001**	30 (46.9)	**12.12**	**≤0.001**	17 (31.5)	**13.36**	**≤0.001**
		TAU	60 (80.0)			50 (80.6)			45 (77.6)			29 (69.1)		
	Does not meet diagnostic criteria	UP	32 (37.6)			35 (58.3)			34 (53.1)			37 (68.5)		
		TAU	15 (20.0)			12 (19.4)			13 (22.4)			13 (30.9)		

Secondary diagnosis	Meets diagnostic criteria	UP	9 (39.1)	**10.42**	**≤0.001**	6 (35.3)	3.44	0.064	5 (33.3)	**4.30**	**0.038**	3 (20.0)	**6.63**	**0.010**
		TAU	13 (92.9)			6 (75.0)			5 (83.3)			6 (75.0)		
	Does not meet diagnostic criteria	UP	14 (60.9)			11 (64.7)			10 (66.7)			12 (80.0)		
		TAU	1 (7.1)			2 (25.0)			1 (16.7)			2 (25.0)		

Note: UP: Unified Protocol; TAU: treatment as usual; T2: 3 months after treatment onset; T3: 6 months after treatment onset; T4: 9 months after treatment onset; T5: 15 months after treatment onset. *p* values < 0.05 are in bold.

**Table 3:** Main effects of the linear mixed models (*N* = 533).

	Primary outcomes												Secondary outcomes														
	BDI-II			ODSIS			BAI			OASIS			Neuroticism			Negative affect			Extraversion			Positive affect			QLI		
	*F*	*p*	Cohen's *d*	*F*	*p*	Cohen's *d*	*F*	*p*	Cohen's *d*	*F*	*p*	Cohen's *d*	*F*	*p*	Cohen's *d*	*F*	*p*	Cohen's *d*	*F*	*p*	Cohen's *d*	*F*	*p*	Cohen's *d*	*F*	*p*	Cohen's *d*
Time	**10.19**	**≤0.001**	**0.28**	**7.55**	**≤0.001**	**0.24**	**7.55**	**≤0.001**	**0.24**	**6.37**	**≤0.001**	**0.22**	**3.83**	**≤0.001**	**0.17**	**12.61**	**≤0.001**	**0.31**	1.02	0.393	0.09	1.64	0.162	0.11	**6.08**	**≤0.001**	**0.21**
Condition	0.55	0.459	0.06	0.64	0.425	0.07	0.78	0.378	0.08	0.120	0.729	0.03	2.76	0.097	0.14	0.01	0.937	0.03	0.33	0.568	0.05	0.01	0.969	0.03	0.03	0.852	0.01
Sessions	1.31	0.253	0.10	1.77	0.183	0.12	0.11	0.744	0.03	3.47	0.063	0.16	0.11	0.738	0.03	1.62	0.204	0.11	0.80	0.372	0.08	**7.76**	**0.005**	**0.24**	0.66	0.416	0.07
Time⁣^∗^Condition	1.20	0.309	0.09	1.74	0.139	0.11	0.86	0.489	0.08	0.503	0.734	0.06	0.61	0.654	0.07	0.11	0.979	0.03	0.60	0.662	0.07	0.37	0.826	0.05	0.30	0.880	0.05
Time⁣^∗^Condition⁣^∗^Sessions	0.93	0.480	0.08	**2.87**	**0.006**	0.15	0.45	0.870	0.06	1.53	0.153	0.11	1.41	0.197	0.10	1.90	0.066	0.12	0.55	0.794	0.06	1.22	0.291	0.10	1.44	0.186	0.10

Note: BDI-II: Beck Depression Inventory; ODSIS: Overall Depression Severity and Impairment Scale; BAI: Beck Anxiety Inventory; OASIS: Overall Anxiety Severity and Impairment Scale; QLI: Quality of Life Index. *p* values < 0.05 are in bold.

**Table 4:** Main effects of the linear mixed models across condition and different treatment periods (*N* = 533).

Main effects														
	Dependent variable		T1	T2	T3	T4	T5	*F*	*p*	Cohen's *d*				
			*M* (SD)	*M* (SD)	*M* (SD)	*M* (SD)	*M* (SD)			T1 to T2	T2 to T3	T3 to T4	T4 to T5	T1 to T5
Primary outcomes	BDI-II	UP	29.00 (12.09)	20.37 (14.02)	18.44 (14.09)	17.55 (14.83)	16.48 (12.81)	**76.98**	**≤0.001**	0.67	0.14	0.06	0.08	1.01
		TAU	28.68 (12.26)	22.15 (12.63)	21.46 (13.95)	18.64 (13.93)	19.37 (14.96)	**42.24**	**≤0.001**	0.53	0.05	0.20	0.05	0.72
	ODSIS	UP	10.58 (4.98)	6.67 (5.62)	6.01 (5.76)	6.02 (6.25)	5.72 (5.48)	**61.04**	**≤0.001**	0.75	0.12	≤0.001	0.05	0.93
		TAU	9.79 (5.48)	8.03 (5.70)	7.64 (5.76)	7.11 (5.34)	6.83 (6.03)	**14.81**	**≤0.001**	0.32	0.07	0.09	0.05	0.53
	BAI	UP	28.06 (13.14)	20.92 (14.12)	18.61 (14.41)	17.56 (14.71)	17.09 (13.91)	**52.77**	**≤0.001**	0.53	0.16	0.07	0.03	0.81
		TAU	27.24 (13.30)	22.99 (14.24)	22.05 (14.26)	20.16 (14.15)	19.48 (14.85)	**21.28**	**≤0.001**	0.31	0.07	0.13	0.05	0.57
	OASIS	UP	11.27 (4.27)	7.58 (5.02)	6.77 (5.28)	6.55 (5.67)	6.30 (5.29)	**67.58**	**≤0.001**	0.81	0.16	0.04	0.04	1.03
		TAU	10.73 (4.58)	8.70 (4.94)	8.66 (5.13)	8.13 (5.03)	7.41 (5.59)	**20.70**	**≤0.001**	0.43	0.01	0.10	0.14	0.69

Secondary outcomes	Neuroticism	UP	33.24 (7.05)	30.05 (7.89)	28.21 (9.03)	27.08 (9.28)	26.69 (8.39)	**41.35**	**≤0.001**	0.43	0.22	0.12	0.04	0.84
		TAU	32.42 (7.57)	30.35 (7.90)	29.58 (7.81)	28.58 (7.77)	27.20 (8.00)	**16.28**	**≤0.001**	0.27	0.10	0.13	0.17	0.68
	Negative affect	UP	29.55 (8.42)	24.49 (8.67)	24.25 (8.38)	22.48 (9.16)	22.10 (8.31)	**29.01**	**≤0.001**	0.59	0.03	0.20	0.04	0.89
		TAU	29.52 (8.38)	25.95 (9.07)	22.3 (7.64)	23.94 (8.18)	24.40 (9.71)	**24.34**	**≤0.001**	0.41	0.43	-0.21	-0.05	0.59
	Extraversion	UP	21.22 (8.30)	23.15 (8.87)	23.93 (9.12)	24.41 (9.32)	24.23 (8.76)	**17.30**	**≤0.001**	-0.23	-0.09	-0.05	0.02	-0.36
		TAU	21.81 (8.42)	22.16 (8.55)	22.07 (8.44)	23.62 (8.34)	23.20 (8.70)	0.66	0.621	0.04	0.01	-0.18	0.05	-0.16
	Positive affect	UP	20.54 (6.84)	23.34 (8.01)	24.25 (8.38)	24.64 (9.21)	26.21 (8.71)	**21.42**	**≤0.001**	-0.38	-0.11	-0.04	-0.17	-0.77
		TAU	20.75 (7.02)	22.39 (7.16)	22.30 (7.64)	22.82 (7.77)	24.17 (8.33)	**5.71**	**≤0.001**	-0.23	0.01	-0.07	-0.17	-0.47
	QLI	UP	4.40 (1.61)	5.38 (1.91)	5.63 (1.83)	5.96 (1.97)	5.97 (1.86)	**57.48**	**≤0.001**	-0.57	-0.13	-0.17	≤0.001	-0.93
		TAU	4.54 (1.56)	5.10 (1.68)	5.24 (1.84)	5.45 (1.85)	5.68 (2.03)	**18.88**	**≤0.001**	-0.35	-0.08	-0.11	-0.12	-0.68

Note: UP: Unified Protocol; TAU: treatment as usual; BDI-II: Beck Depression Inventory; ODSIS: Overall Depression Severity and Impairment Scale; BAI: Beck Anxiety Inventory; OASIS: Overall Anxiety Severity and Impairment Scale; QLI: Quality of Life Index; T1: pretreatment; T2: 3 months after treatment onset; T3: 6 months after treatment onset; T4: 9 months after treatment onset; T5: 15 months after treatment onset. *p* values < 0.05 are in bold.

**Table 5:** ANOVA between treatment conditions throughout the intervention with intention-to-treat participants and per-protocol noninferiority analysis.

	Dependent variable	T1			T2			T3			T4			T5			Per-protocol non-inferiority analysis			
																	T1 to T2		T1 to T5	
		*F*	*p*	Cohen's *d*	*F*	*p*	Cohen's *d*	*F*	*p*	Cohen's *d*	*F*	*p*	Cohen's *d*	*F*	*p*	Cohen's *d*	*W*	*p*	*W*	*p*
Primary outcomes	BDI-II	0.45	0.505	0.06	0.89	0.347	0.10	3.80	0.052	0.22	0.34	0.559	0.08	1.69	0.195	0.20	**-2.13**	**0.017**	**-2.08**	**0.020**
	ODSIS	3.51	0.062	0.16	**4.85**	**0.028**	**0.24**	**7.06**	**0.008**	0.30	2.26	0.134	0.19	1.12	0.291	0.16	**-5.54**	**≤0.001**	**-2.53**	**0.006**
	BAI	0.81	0.368	0.08	1.48	0.225	0.13	**4.61**	**0.033**	0.04	1.80	0.181	0.17	0.74	0.391	0.13	**-2.73**	**0.003**	-1.51	0.067
	OASIS	3.60	0.058	0.16	3.55	0.060	0.20	**10.50**	**≤0.001**	**0.37**	**5.06**	**0.025**	0.29	0.97	0.326	0.15	**-4.93**	**≤0.001**	**-2.40**	**0.009**

Secondary outcomes	Neuroticism	2.28	0.132	0.13	0.01	0.937	0.01	1.63	0.203	0.15	1.04	0.309	0.13	0.02	0.897	0.02	**-2.70**	**0.004**	-1.29	0.100
	Negative affect	0.32	0.571	0.05	1.10	0.294	0.11	**6.48**	**0.011**	0.29	1.01	0.315	0.13	1.59	0.210	0.19	**-2.57**	**0.005**	-1.07	0.143
	Extraversion	0.89	0.346	0.08	0.72	0.397	0.09	3.08	0.080	0.20	0.36	0.550	0.08	0.31	0.578	0.08	**2.88**	**0.002**	**3.00**	**0.002**
	Positive affect	0.18	0.669	0.04	0.71	0.401	0.09	3.02	0.084	0.20	2.34	0.127	0.20	2.34	0.128	0.23	**1.98**	**0.024**	**3.24**	**≤0.001**
	QLI	1.60	0.207	0.11	1.19	0.276	0.12	**4.81**	**0.029**	**0.25**	**4.23**	**0.041**	0.27	0.83	0.364	0.14	**2.41**	**0.008**	1.29	0.100

Note: UP: Unified Protocol; TAU: treatment as usual; BDI-II: Beck Depression Inventory; ODSIS: Overall Depression Severity and Impairment Scale; BAI: Beck Anxiety Inventory; OASIS: Overall Anxiety Severity and Impairment Scale; QLI: Quality of Life Index; T1: pretreatment; T2: 3 months after treatment onset; T3: 6 months after treatment onset; T4: 9 months after treatment onset; T5: 15 months after treatment onset; *W*: Welch's *t*-test. The value of *W* is negative or positive when the test is applied to the lower bound or upper bound, respectively. *p* values < 0.05 are in bold.

**Table 6:** Post hoc analyses for the Time⁣^∗^Condition⁣^∗^Sessions effects (*N* = 533).

Main effects														
Dependent variable		Sessions	T1	T2	T3	T4	T5	*F*	*p*	Cohen's *d*				
			*M* (SD)	*M* (SD)	*M* (SD)	*M* (SD)	*M* (SD)			T1 to T2	T2 to T3	T3 to T4	T4 to T5	T1 to T5
ODSIS	UP	8 to 12	12.61 (2.80)	8.71 (5.57)	5.45 (5.75)	6.75 (6.87)	6.22 (6.33)	**11.10**	**≤0.001**	0.95	0.58	-0.21	0.08	1.57
		>12	10.33 (4.57)	7.40 (5.37)	6.68 (5.88)	7.22 (6.12)	6.67 (5.27)	**15.85**	**≤0.001**	0.60	0.13	-0.09	0.09	0.77
	TAU	<8	10.85 (4.81)	9.95 (5.81)	9.15 (5.65)	7.25 (5.14)	7.07 (6.31)	**8.33**	**≤0.001**	0.17	0.14	0.35	0.03	0.73
		8 to 12	8.84 (5.39)	8.61 (6.46)	8.68 (5.34)	9.31 (6.13)	8.52 (6.15)	0.088	0.986	0.04	0.01	-0.11	0.13	0.06

Note: UP: Unified Protocol; TAU: treatment as usual; ODSIS: Overall Depression Severity and Impairment Scale. T1: pretreatment; T2: 3 months after treatment onset; T3: 6 months after treatment onset; T4: 9 months after treatment onset; T5: 15 months after treatment onset. *p* values < 0.05 are in bold. We only include here the significant effect of Time⁣^∗^Condition⁣^∗^Sessions (Table 3).

## Data Availability

The data that support the findings of this study are available from the corresponding author, Jorge Osma (osma@unizar.es), upon reasonable request.
